# Branched multipeptide immunotherapy for glioblastoma using human leukocyte antigen-A*0201-restricted cytotoxic T-lymphocyte epitopes from ERBB2, BIRC5 and CD99

**DOI:** 10.18632/oncotarget.10495

**Published:** 2016-07-08

**Authors:** Young-Hee Kim, Thi-Anh-Thuy Tran, Hyun-Ju Lee, Sook-In Jung, Je-Jung Lee, Wool-Youl Jang, Kyung-Sub Moon, In-Young Kim, Shin Jung, Tae-Young Jung

**Affiliations:** ^1^ Brain Tumor Research Laboratory, Chonnam National University Hwasun Hospital, Chonnam, Republic of Korea; ^2^ Research Center for Cancer Immunotherapy, Chonnam National University Hwasun Hospital, Chonnam, Republic of Korea; ^3^ Department of Internal Medicine, Chonnam National University Medical School, Gwangju, Republic of Korea; ^4^ Department of Hematology-Oncology, Chonnam National University Hwasun Hospital, Chonnam, Republic of Korea; ^5^ Department of Neurosurgery, Chonnam National University Hwasun Hospital & Medical School, Gwangju, Republic of Korea

**Keywords:** multipeptide, immunotherapy, glioblastoma, dendritic cell

## Abstract

We investigated the use of cytotoxic T-lymphocyte (CTL) epitopes in peptide immunotherapy for glioblastoma. Three peptides (ERBB2, BIRC5 and CD99) were selected based on their peptide-T2 cell binding affinities and combined in a multipeptide cocktail or a branched multipeptide synthesized with mini-polyethylene glycol spacers. Dendritic cells (DCs) pulsed with the multipeptide cocktail or branched multipeptide were compared based on their immunophenotype and cytokine secretion. FACS analysis of alpha-type 1 polarized dendritic cells (αDC1s) revealed that both groups highly expressed CD80, CD83 and CD86, indicating that both treatments efficiently generated mature αDC1s with the expected phenotype. Production of IL-12p70, IL-12p40 and IL-10 also increased upon αDC1 maturation in both groups. CTLs stimulated by either αDC1 group (“DC-CTLs”) included numerous IFN-γ-secreting cells against T2 cells loaded with the corresponding multipeptides. Large numbers of IFN-γ-secreting cells were observed when human glioblastoma cell lines and primary cells were treated with multipeptide-pulsed DC-CTLs. Both multipeptide-pulsed DC-CTL groups exhibited cytotoxic activity of 40-60% against the U251 cell line and 60-80% against primary cells. Branched multipeptide from ERBB2, BIRC5 and CD99 stably bound with T2 cells, and its cytotoxicity toward target cells was similar to that of the multipeptide cocktail. Thus, branched multipeptides could be promising candidates for immunotherapeutic glioblastoma treatment.

## INTRODUCTION

Gliomas are the most common primary tumors of the central nervous system [1, 2]. Despite advances in conventional treatments such as surgical resection, radiation therapy and chemotherapy, the prognosis for most patients with glioblastomas is poor. Even with intensive treatment, glioblastomas frequently recur due to continued growth of the residual microscopic disease located beyond surgical resection margins [3].

In recent years, numerous attempts have been made to develop immunotherapies and adjuvant therapies to more effectively treat glioblastomas [3–6]. One promising modality for glioblastoma treatment is specific immunotherapy using dendritic cells (DCs) [4, 7]. This modality requires information about the target antigens and their epitope peptides that are recognized by T cells. A major challenge for *in vitro* induction of glioblastoma-reactive cytotoxic T-lymphocytes (CTLs) for adoptive immunotherapy is the identification of major histocompatibility complex (MHC) class I-restricted CTL epitopes derived from glioblastoma tumor-associated antigens (TAAs). DCs are potent antigen-presenting cells (APCs), which are highly efficient in antigen presentation and the stimulation of T lymphocytes [8]. However, DC immunotherapy has some disadvantages, such as the limited availability of materials for designing vaccines, the costly and labor-intensive preparation, and the requirement of a reliable laboratory marker. This situation has stimulated interest in exploring the usefulness of peptide immunotherapy for patients with malignant glioblastomas [6, 9–11]. The advantages of synthetic CTL epitope peptides include their ease of production, pathogen-free nature and chemical stability.

Previous studies have discussed brain TAAs and their peptides, but as human brain tumors express a wide variety of TAAs, there is still a need to identify therapeutically useful glioblastoma-TAAs and their epitope peptides [12–16]. In our previous study, we detected positive expression of v-erb-b2 erythroblastic leukemia viral oncogene homolog-2 (ERBB2), baculoviral IAP repeat containing-5 (BIRC5) and the glycoprotein CD99 in most glioblastoma tissues, and negative expression in normal brain tissues [17]. As these TAAs are highly expressed in glioblastoma tissues and cell lines, they could be the targets of synthetic multipeptide immunotherapy.

Synthetic CTL epitope peptides such as multipeptides have greater molecular weights and immunogenicity than their corresponding peptides [10, 18]. In this study, we designed a branched multipeptide by using mini-polyethylene glycol (mini-PEG) spacers. The attachment of mini-PEGs to peptides increases their metabolic half-lives, lowers their non-specific binding and shields them from proteolytic enzymes [19]. Multipeptides can induce human leukocyte antigen (HLA)-restricted and tumor-reactive CTLs. HLA-A*0201 is highly frequent in the populations of Northern Asia and North America, and is the most common type of HLA in Korea (16.5%) [20].

We selected three peptides derived from the brain TAAs of ERBB2, BIRC5 and CD99 with an HLA-A*0201-binding epitopes recognized by CTLs, investigated the potential of peptide immunotherapy for glioblastoma by using CTLs generated by branched multipeptide-pulsed alpha-type 1 polarized dendritic cells (αDC1s), and compared this immunotherapy with a multipeptide cocktail of ERBB2, BIRC5 and CD99.

## RESULTS

### Selection and synthesis of ERBB2, BIRC5 and CD99 peptides

**Figure 1:**
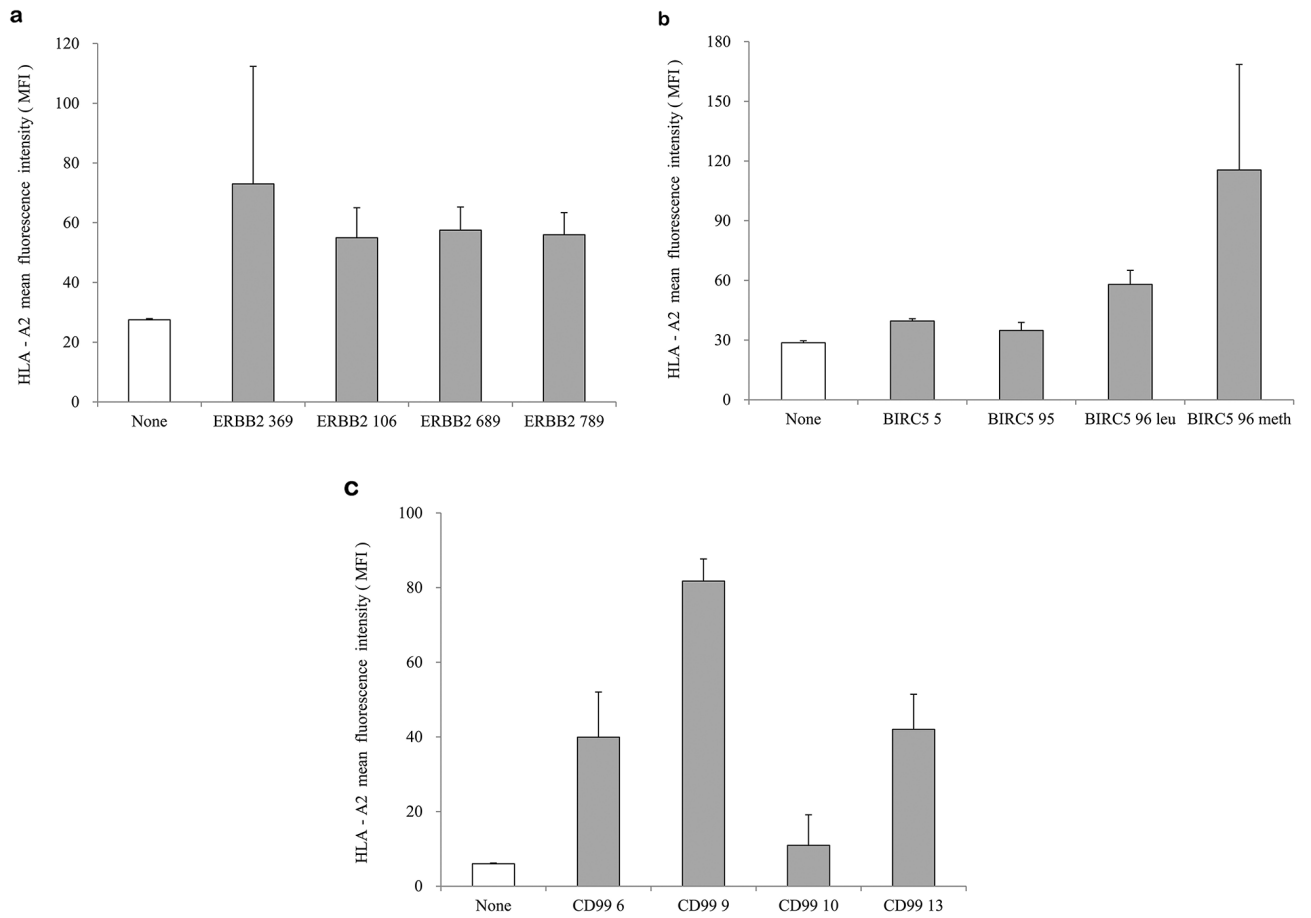
Peptide-binding affinity and selection of peptide candidates **a.** ERBB2 peptides, **b.** BIRC5 peptides, **c.** CD99 peptides. ERBB2_369_ (KIFGSLAFL), BIRC5_96methionine_ (LMLGEFLKL) and CD99_9_ (LLLFGLLGV) were selected as HLA-A*0201-specific peptides with high scores in the peptide-T2 binding assay. The concentration of each peptide was 20 μg/mL. The values represent the mean MFI ± SE of three separate experiments.

We measured the binding affinity of HLA-A*0201-specific peptides in a peptide-T2 binding assay, and selected peptides (20μg/mL each) with high scores for further analysis: ERBB2_369_ (KIFGSLAFL), BIRC5_96methionine_ (LMLGEFLKL) and CD99_9_ (LLLFGLLGV) (Figure 1). Furthermore, we used mini-PEG spacers to create their corresponding branched multipeptide, which was designated as (ERBB2 - mini PEG2 - K {BIRC5 - mini PEG2 - K [CD99 - mini PEG2]}) [21].

### HLA-A02 binding affinity and stability of the multipeptide cocktail and branched multipeptide

**Figure 2:**
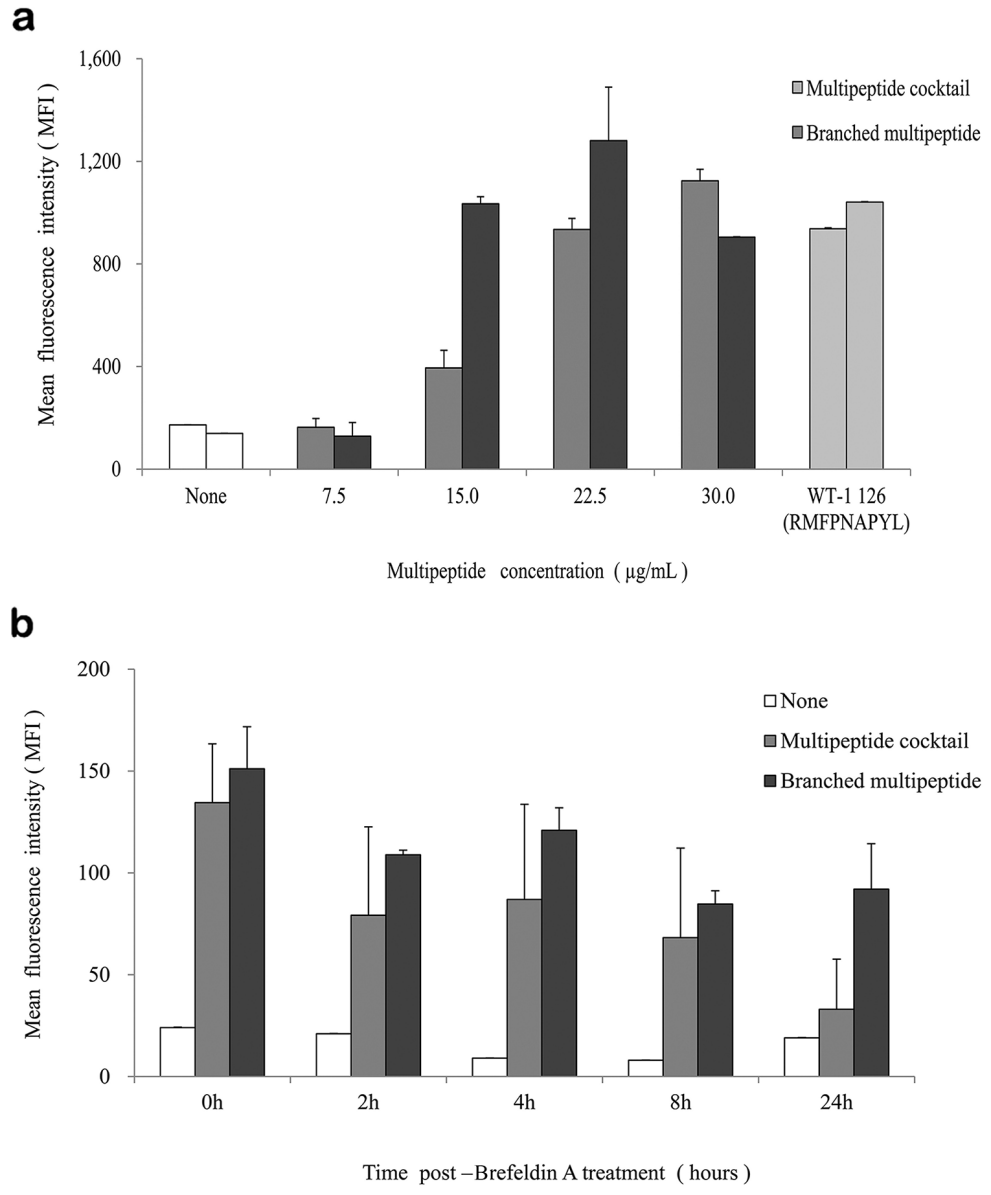
HLA-A02 binding affinity and stability of the multipeptide cocktail and branched multipeptide **a.** Binding affinity: The multipeptide cocktail dose-dependently increased the HLA-A02 mean MFI in T2 cells. In cells treated with the branched multipeptide, the mean MFI increased until the total peptide concentration reached 22.5 μg/mL (7.5 μg/mL/peptide), which was selected as the optimal peptide concentration. **b.** Binding stability: While the stability of the branched multipeptides was maintained up to 24 hours post-BFA treatment, the stability of the multipeptide cocktail was only maintained up to 8 hours post-BFA treatment. The values represent the mean MFI ± SE of three separate experiments.

The T2 cell line was used to evaluate the binding affinity of the HLA-A*0201-specific multipeptide cocktail and the branched multipeptide. In the peptide-binding assay, the multipeptide cocktail dose-dependently increased the HLA-A02 mean fluorescence intensity (MFI) in T2 cells (0, 7.5, 15.0, 22.5, 30.0 μg/mL) (Figure 2a). However, in cells treated with the branched multipeptide, the mean MFI increased until the total peptide concentration reached 22.5 μg/mL (7.5 μg/mL/peptide) and decreased thereafter. Therefore, 22.5 μg/mL was selected as the optimal peptide concentration for evaluating HLA-A02 binding stability. Flow cytometric analysis revealed a difference in the peptide-binding stabilities of the multipeptide cocktail and the branched multipeptide. Both multipeptides tended to destabilize over time; however, the stability of the branched multipeptide was maintained for up to 24 hours post-Brefeldin A (BFA) treatment, while the stability of the multipeptide cocktail was only maintained for up to 8 hours post-BFA treatment (Figure 2b). Moreover, at 24 hours post-BFA treatment, we observed a decrease in HLA-A02-specific affinity.

### Characterization of αDC1s pulsed with the multipeptide cocktail or the branched multipeptide

**Figure 3:**
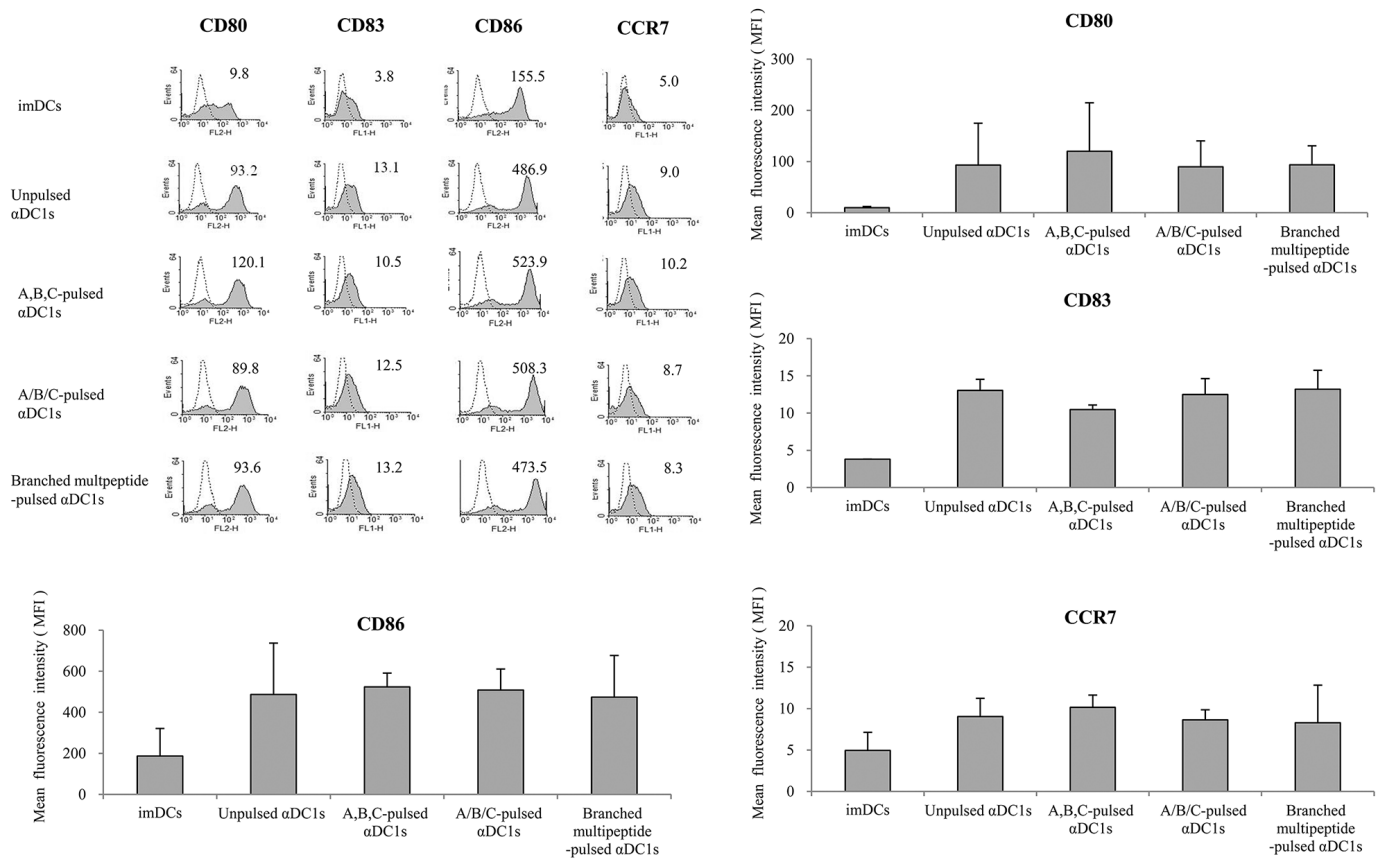
Immunophenotype of DCs pulsed with multipeptides for the cell surface markers (CD80, CD83, CD86, CCR7) No significant differences were found in the expression of CD80, CD83, CD86, or CCR7 (relative to that of immature DCs) between the four αDC1 populations (Unpulsed, A,B,C-pulsed, A/B/C-pulsed, Branched multipeptide-pulsed). Results are reported as mean MFI values ± SE of three separate experiments. (imDCs: immature DCs; Unpulsed αDC1s: peptide-unpulsed αDC1s; A,B,C-pulsed αDC1s: ERBB2, BIRC5 and CD99 individual peptide-pulsed αDC1s; A/B/C-pulsed αDC1s: mixed ERBB2, BIRC5 and CD99 peptide-pulsed αDC1s; Branched multipeptide-pulsed αDC1s: branched multipeptide-pulsed αDC1s).

We used flow cytometric analysis to determine the phenotypes of αDC1s pulsed with the multipeptide cocktail or the branched multipeptide, as well as that of unpulsed cells. ERBB2, BIRC5 and CD99 individual peptide-pulsed αDC1s were termed as “A,B,C-pulsed αDC1s,” while mixed ERBB2, BIRC5 and CD99 peptide-pulsed αDC1s were termed as “A/B/C-pulsed αDC1s,” and branched multipeptide-pulsed αDC1s were termed as “Branched multipeptide-pulsed αDC1s” (A: ERBB2, B: BIRC5, C: CD99). As shown in Figure 3, the above-mentioned four αDC1 populations (Unpulsed, A,B,C-pulsed, A/B/C-pulsed, Branched multipeptide-pulsed) displayed a characteristic set of mature αDC1 surface markers not displayed by immature DCs. No significant differences were found in the expression of CD80, CD83, CD86, or CCR7 among the four αDC1 populations. Thus, αDC1s pulsed with the multipeptide cocktail or branched multipeptide efficiently generated mature αDC1s with the expected phenotype.

**Figure 4:**
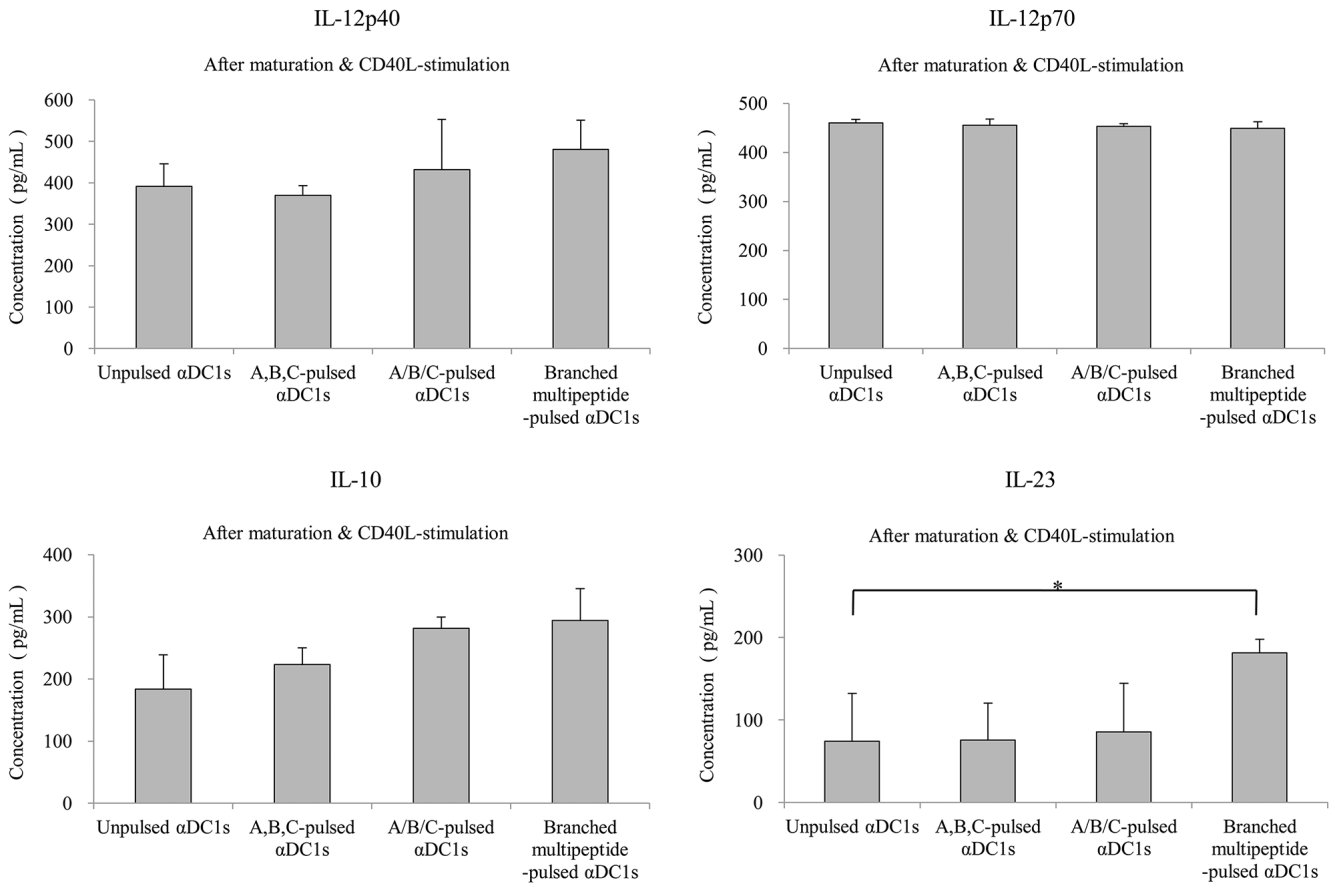
Cytokine secretion of αDC1s pulsed with multipeptides Production of IL-12p40, IL-12p70, IL-10 and IL-23. Cytokines secreted into the culture supernatants were measured by ELISA after stimulation with CD40 ligand-transfected J558 cells. The production of IL-12p40, IL-12p70, and IL-10 did not differ significantly among unpulsed αDC1s, αDC1s pulsed with the multipeptide cocktail and those pulsed with branched multipeptide. Branched multipeptide-pulsed mature αDC1s produced higher levels of IL-23 than unpulsed and multipeptide cocktail-pulsed αDC1s (*p* = 0.047). The results shown are from the triplicate culture from three independent experiments and are expressed as the mean ± SE. (Unpulsed αDC1s: peptide-unpulsed αDC1s; A,B,C-pulsed αDC1s: ERBB2, BIRC5 and CD99 individual peptide-pulsed αDC1s; A/B/C-pulsed αDC1s: mixed ERBB2, BIRC5 and CD99 peptide-pulsed αDC1s; Branched-multipeptide-pulsed αDC1s: branched-multipeptide-pulsed αDC1s) (*: p < 0.05).

We then measured IL-12p40, IL-12p70, IL-10 and IL-23 production by unpulsed αDC1s and mature αDC1s pulsed with the multipeptide cocktail or the branched multipeptide (Figure 4). Mature αDC1s produced higher levels of IL-12p40 and IL-12p70 than immature DCs. After subsequent stimulation with CD40L-transfected J558 cells, the mature αDC1s pulsed with the multipeptide cocktail or the branched multipeptide and unpulsed αDC1s produced similar levels of IL-12p40 and IL-12p70. Similarly, the production of the inhibitory cytokine IL-10 by mature αDC1s increased after CD40L stimulation. The production of IL-12p40, IL-12p70 and IL-10 did not differ significantly among unpulsed αDC1s, αDC1s pulsed with the multipeptide cocktail and those pulsed with branched multipeptide (*p* = 0.322, 0.827 and 0.099, respectively). IL-23 production by αDC1s did not increase significantly during the maturation phase. After subsequent stimulation with CD40L-transfected J558 cells, branched multipeptide-pulsed mature αDC1s produced greater levels of IL-23 than unpulsed and multipeptide cocktail-pulsed αDC1s. The difference in IL-23 production was statistically significant (*p* = 0.047).

### IFN-γ secretion by multipeptide cocktail- and branched multipeptide-specific CTLs against multipeptide-pulsed T2 cells

**Figure 5:**
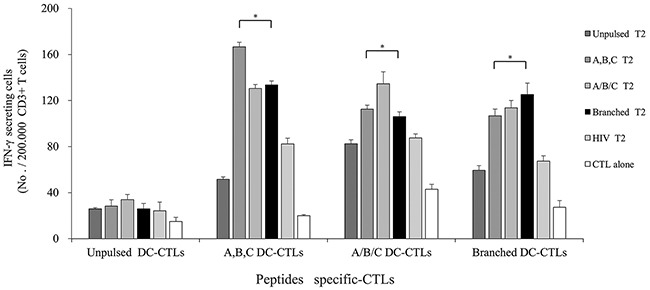
IFN-γ ELISPOT assay of multipeptide-specific CTLs against multipeptide-pulsed T2 cells IFN-γ secretion of A,B,C DC-CTLs, A/B/C DC-CTLs and Branched DC-CTLs. Multipeptide-pulsed T2 cells were used as the target cells, and unpulsed and HIV peptide-pulsed T2 cells were used as negative controls. The E/T ratio was 10:1. A,B,C DC-CTLs, A/B/C DC-CTLs and Branched DC-CTLs more specifically produced IFN-γ against the A,B,C T2, A/B/C T2 and Branched T2 target cells, respectively. The specificity of the CTLs was statistically significant (*p* = 0.002, 0.011 and 0.022, respectively). (A,B,C DC-CTLs: ERBB2, BIRC5 and CD99 individual peptide-pulsed DCs stimulated with CD3^+^ T cells; A/B/C DC-CTLs: mixed ERBB2, BIRC5 and CD99 peptide-pulsed DCs stimulated with CD3^+^ T cells; Branched DC-CTLs: branched-multipeptide-pulsed DCs stimulated with CD3^+^ T cells; Unpulsed T2: peptide-unpulsed T2 cells; A,B,C T2: T2 cells individually pulsed with the ERBB2, BIRC5 and CD99 peptides; A/B/C T2: T2 cells pulsed with mixed ERBB2, BIRC5 and CD99 peptides; Branched T2: branched multipeptide-pulsed T2 cells; HIV T2: HIV peptide-pulsed T2 cells) (*: p < 0.05).

Multipeptide cocktail-pulsed T2 cells (A,B,C T2, A/B/C T2), branched multipeptide-pulsed T2 cells (Branched T2), unpulsed T2 cells (Unpulsed T2), and HIV peptide-pulsed T2 cells (HIV T2) were used as target cells. Three effector CTL groups (A,B,C DC-CTLs, A/B/C DC-CTLs and Branched DC-CTLs) were compared for their ability to recognize T2 cells pulsed with the multipeptide cocktail or branched multipeptide. As shown in Figure 5, the CTLs generated by αDC1s pulsed with the multipeptide cocktail or branched multipeptide included a large number of IFN-γ-secreting cells, and were more potent than the CTLs stimulated by unpulsed αDC1s at an effector/target (E/T) ratio of 10:1. A,B,C DC-CTLs, A/B/C DC-CTLs and Branched DC-CTLs more specifically produced IFN-γ against the A,B,C T2, A/B/C T2 and Branched T2 target cells, respectively. The specificity of the CTLs was statistically significant (*p* = 0.002, 0.011 and 0.022, respectively).

### IFN-γ secretion by multipeptide cocktail- and branched multipeptide-specific CTLs against human glioblastoma cell lines and primary glioblastoma cells

**Figure 6:**
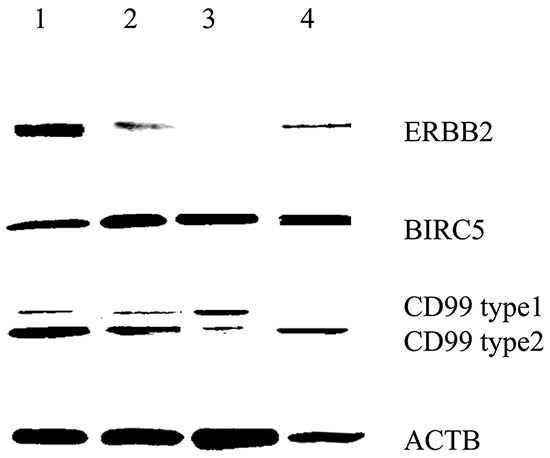
BIRC5, CD99 and ERBB2 expression of human glioblastoma cell lines and primary glioblastoma cells by Western blot All cells were HLA-A2 positive. The U87 and U251 cell lines and primary cells positively expressed ERBB2, BIRC5 and CD99. The U343 cell line positively expressed BIRC5 and CD99. (Lane 1: U87 cell line, Lane 2: U251 cell line, Lane 3: U343 cell line, Lane 4: Primary glioblastoma cells).

**Figure 7:**
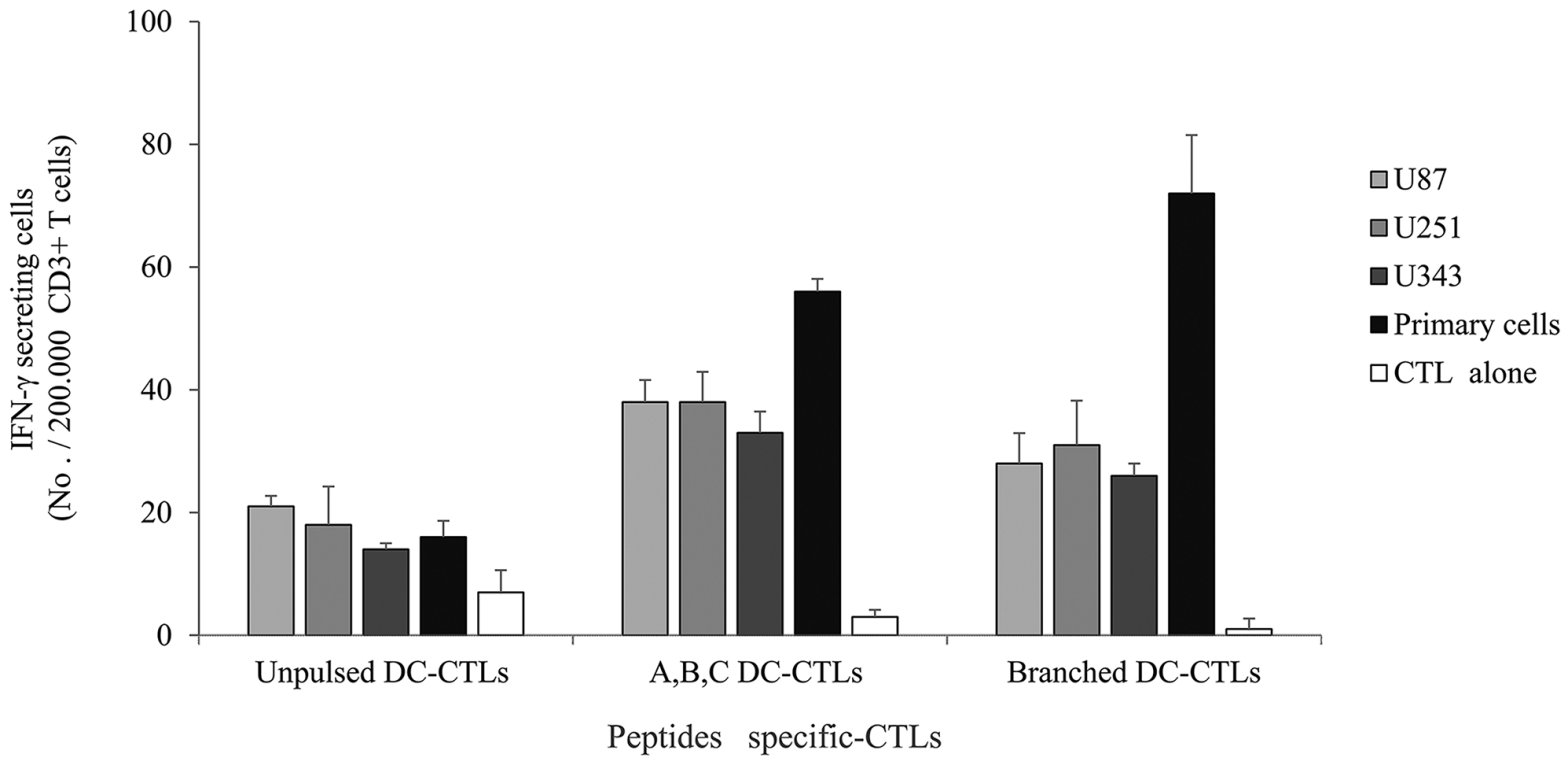
IFN-γ ELISPOT assay of multipeptide-specific CTLs against glioblastoma cell lines and primary glioblastoma cells IFN-γ secretion of A,B,C DC-CTLs and Branched DC-CTLs. A,B,C DC-CTLs and Branched DC-CTLs produced a higher number of IFN-γ spots against glioblastoma cell lines and primary glioblastoma cells than Unpulsed DC-CTLs. A,B,C DC-CTLs and Branched DC-CTLs exhibited a specific response, and clearly differed from Unpulsed DC-CTLs in their ability to produce IFN-γ against the target cells. (Unpulsed DC-CTLs: peptide-unpulsed DCs stimulated with autologous CD3^+^ T cells; A,B,C DC-CTLs: ERBB2, BIRC5 and CD99 individual peptide-pulsed DCs stimulated with CD3^+^ T cells; Branched DC-CTLs: branched-multipeptide-pulsed DCs stimulated with CD3^+^T cells; U87: U87 glioblastoma cell line; U251: U251 glioblastoma cell line; U343: U343 glioblastoma cell line; Primary cells: primary glioblastoma cells; CTL alone: no target cells).

We used HLA-A2-positive human glioblastoma cell lines and primary cells for additional experiments. Western blotting revealed that the U87 and U251 cell lines and primary cells positively expressed ERBB2, BIRC5 and CD99 (Figure 6). The U343 cell line positively expressed BIRC5 and CD99. IFN-γ secretion against glioblastoma cell lines and primary glioblastoma cells was studied at an E/T ratio of 10:1 (Figure 7). This experiment was performed with multipeptide cocktail-specific CTLs (A,B,C DC-CTLs) and branched multipeptide-specific CTLs (Branched DC-CTLs), with the U87, U251 and U343 cell lines and primary glioblastoma cells as the target cells. Unpulsed DC-CTLs were used as the negative control. A,B,C DC-CTLs and Branched DC-CTLs produced a higher number of IFN-γ spots than Unpulsed DC-CTLs against glioblastoma cell lines and primary glioblastoma cells. A,B,C DC-CTLs and Branched DC-CTLs exhibited a specific response, and clearly differed from Unpulsed DC-CTLs in their ability to produce IFN-γ against the target cells.

### Lactate dehydrogenase release induced by multipeptide cocktail- and branched multipeptide-specific CTLs in the U251 glioblastoma cell line and primary glioblastoma cells

**Figure 8:**
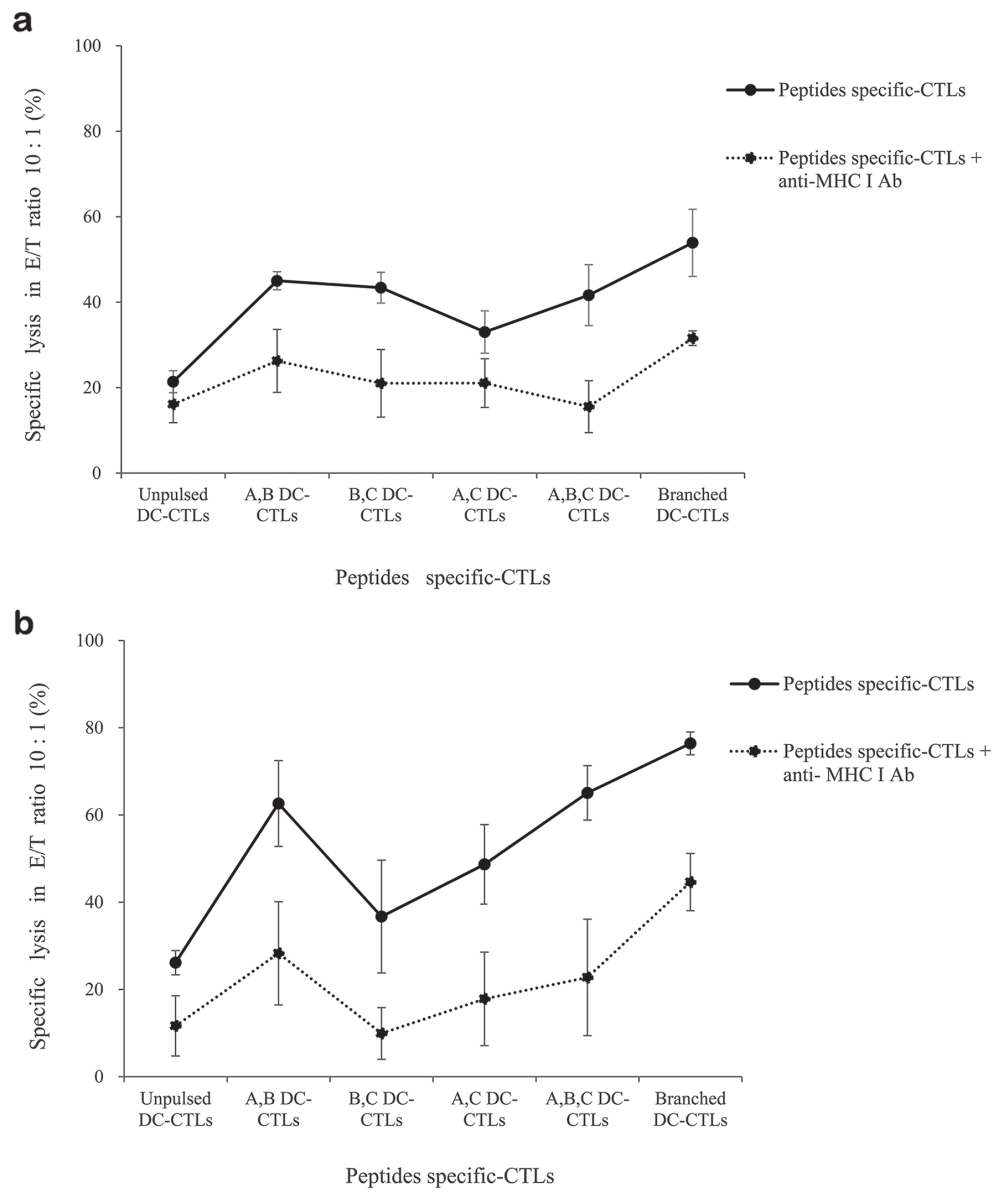
Lactate dehydrogenase release induced by multipeptide cocktail- and branched multipeptide-specific CTLs in the U251 glioblastoma cell line and primary glioblastoma cells LDH release by the U251 cell line **a.** and primary glioblastoma cells **b.** For the U251 cell line, A,B DC-CTLs, B,C DC-CTLs, A,B,C DC-CTLs and Branched DC-CTLs exhibited cytotoxic activity of 40-60%. For primary cells, A,B DC-CTLs, A,B,C DC-CTLs and Branched DC-CTLs exhibited cytotoxic activity of 60-80%. Incubation with an MHC class I antibody reduced the cytotoxic activity to approximately 20% cell lysis in both types of cells. (A: ERBB2, B: BIRC5, C: CD99. A,B DC-CTLs: ERBB2 and BIRC5 individual peptide-pulsed DCs stimulated with CD3^+^ T cells; B,C DC-CTLs: BIRC5 and CD99 individual peptide-pulsed DCs stimulated with CD3^+^ T cells; A,C DC-CTLs: ERBB2 and CD99 individual peptide-pulsed DCs stimulated with CD3^+^ T cells; A,B,C DC-CTLs: ERBB2, BIRC5 and CD99 individual peptide-pulsed DCs stimulated with CD3^+^ T cells; Branched DC-CTLs: branched-multipeptide-pulsed DCs stimulated with CD3^+^ T cells).

We tested the ability of multipeptide cocktail- and branched multipeptide-specific CTLs to kill U251 and primary cells by using a standard LDH release assay at an E/T ratio of 10:1. For the U251 cell line, A,B DC-CTLs, B,C DC-CTLs, A,B,C DC-CTLs and Branched DC-CTLs exhibited cytotoxic activities of 40-60% (Figure 8a). For primary cells, A,B DC-CTLs, A,B,C DC-CTLs and Branched DC-CTLs exhibited cytotoxic activities of 60-80% (Figure 8b). Incubation with an MHC class I antibody reduced cytotoxic activity to approximately 20% cell lysis in both the U251 cell line and primary cells, confirming that the cytotoxicity was MHC class I-specific.

## DISCUSSION

A broad range of immunological defects have been documented for glioblastoma patients, including reduced T cell numbers, impaired T cell responsiveness, and defective signaling after T cell receptor (TCR)/CD3 stimulation [22]. Even with these limitations, DC-based brain tumor immunotherapy has been reported to be successful in animal models as well as in humans, and DC vaccination has been found to be safe and not associated with autoimmunity [7, 23, 24]. Several vaccines composed of peptide-pulsed DCs have been designed based on prior identification of CTL-defined synthetic peptide epitopes. However, DC immunotherapy has some disadvantages, such as the limited availability of materials for designing vaccines, the labor-intensive preparation, and the requirement of a reliable laboratory marker.

A recent study demonstrated the clinical benefit of peptide immunotherapy [6, 25]. The peptide vaccine was affordable, easy to manufacture, and customizable to individual patients' antigen profiles. Synthetic long peptides resist protease activity, prolong presentation to APCs, enhance immunogenicity, and induce both T-cytotoxic and T-helper responses. Synthetic multipeptides have greater molecular weights and immunogenicity than their corresponding peptides [18]. In the present study, we designed a peptide immunotherapy for glioblastoma using ERBB2, BIRC5 and CD99 peptides as TAAs [17]. Previous studies have indicated that BIRC5 and ERBB2 protein expression is absent from the normal brain, while CD99 expression is relatively low (www.proteinatlas.org) [26]. Among other normal tissues, the oral mucosa, esophagus, genital organs and skin express medium levels of the BIRC5 protein. ERBB2 expression is medium in the appendix, heart muscle, skeletal muscle, nasopharynx, bronchus, bladder, breast, female genitalia and skin. CD99 expression is high in the bone marrow, spleen, nasopharynx, pancreas, esophagus and genital organs, so treatments targeting this protein might have more side effects than those targeting the previous two.

TAA peptides, along with the MHC class I complex, bind to TAA-specific TCRs on CD8^+^ cells and activate the T cells. Thus, epitopes that are correctly presented by tumor cells and are recognized by specific T cells can elicit potent anti-tumor responses. As mentioned above, the branched multipeptide in this study was synthesized with mini-PEG spacers. The attachment of mini-PEGs to peptides increases their metabolic half-lives, lowers their non-specific binding and shields them from proteolytic enzymes and pharmacokinetics [21]. On the other hand, mini-PEGs may interfere with receptor binding [21].

A major challenge in the induction of antigen-specific CTLs is the identification of MHC class I-restricted CTL epitopes derived from glioblastoma TAAs [12, 15]. Identification of CTL epitopes from these antigens is a critical step in the development of peptide immunotherapies for cancer. In the present study, we investigated the possibility of using peptide immunotherapy for glioblastoma by using ERBB2-, BIRC5- and CD99-specific CTLs generated by multipeptide-pulsed DCs. We identified and synthesized ERBB2, BIRC5 and CD99 peptides as well as their corresponding branched multipeptide, and then generated CTLs by using DCs pulsed with these peptides as APCs. These CTLs specifically recognized ERBB2, BIRC5 and CD99 multipeptide-pulsed T2 cells and were able to lyse target T2 cells positive for ERBB2, BIRC5 and CD99 efficiently. However, T2 cells can present an unnaturally high number of epitopes upon exposure to a high peptide concentration, so their use may lead to an inaccurate assessment of T-cell sensitivity for immunotherapeutic applications [27]. Therefore, glioblastoma cell lines and primary glioblastoma cells were also used as target cells. As shown in Figure 7, CTLs stimulated by ERBB2, BIRC5 and CD99 peptide-pulsed DCs could recognize these three peptides and produce IFN-γ when the peptides were naturally presented by glioblastoma cell lines and primary glioblastoma cells in the context of HLA-A*0201, and could kill ERBB2-, BIRC5- and CD99-positive tumor cells in the LDH cytotoxicity assay. CTLs stimulated with branched multipeptide-pulsed DCs exhibited comparable cytotoxicity to those stimulated with different combinations of the three peptide-pulsed DCs.

In summary, we have described a branched multipeptide vaccine capable of stimulating a response to multiple CD8^+^ T cell epitopes from glioblastoma-TAAs such as ERBB2, BIRC5 and CD99. Finally, through this work, we confirmed that the branched multipeptide of ERBB2, BIRC5 and CD99 stably bound to T2 cells and was similar to the multipeptide cocktail in its cytotoxicity. Branched multipeptide therapy can thus be considered a useful immunotherapeutic modality for glioblastoma.

## MATERIALS AND METHODS

### Target cells

The T2 cell line, a human B and T cell hybrid expressing HLA-A2 molecules, was obtained from the Korean Cell Line Bank, Seoul, Korea. T2 cells were cultured in RPMI-1640 supplemented with 10% fetal bovine serum (FBS) and 1% penicillin–streptomycin (PS) (all from Gibco-BRL, Grand Island, NY, USA).

The human glioblastoma cell lines U87, U251 and U343 were obtained from the Korean Cell Line Bank (Seoul, Korea) and the Brain Tumor Research Center, University of California (San Francisco, CA, USA). Brain tissue specimens were obtained from patients undergoing surgery in the Department of Neurosurgery at Chonnam National University Hwasun Hospital. The fresh tumor samples were washed, fractionated in PBS, and enzymatically dissociated with 0.3% collagenase. The primary glioblastoma cells were then resuspended. All these cells were HLA-A2-positive. These cells were routinely grown in Dulbecco's Modified Eagle's Medium (DMEM; Gibco-BRL, Gaithersburg, MD, USA) supplemented with 10% FBS and 1% PS at 37°C in a humidified atmosphere containing 95% air and 5% CO_2_.

### ERBB2, BIRC5 and CD99 expression by western blot

The U87, U251 and U343 cell lines were homogenized with lysis buffer [50 mM Tris (pH 8.0), 5 mM EDTA, 150 mM sodium chloride, 0.5% deoxycholic acid, 0.1% sodium dodecyl sulfate (SDS), 1% NP-40, 1 mM phenyl methane-sulfonyl fluoride (PMSF) and 1 mg/mL protease inhibitor cocktail]. Protein concentrations were determined with a Bio-Rad protein assay kit (Bio-Rad, Hercules, CA, USA). Subsequently, 50 μg of whole cell lysate was separated by 15% SDS-polyacrylamide gel electrophoresis (PAGE) and transferred to a polyvinylidene difluoride membrane (Pall Corporation, Ann Arbor, MI, USA). The membrane was then incubated for two hours at room temperature in a solution containing TBST [10 mM TrisCl (pH 8.0), 150 mM NaCl and 0.05% Tween 20] supplemented with 5% non-fat dry milk, and was probed overnight at 4°C with antibodies to ERBB2 (Cell Signaling, USA), BIRC5 (SantaCruz Biotechnology, Santa Cruz, CA, USA), or CD99 (AbCam, Cambridge, United Kingdom). The bound antibodies were visualized with goat anti-rabbit (1:40,000; Jackson Immunoresearch, West Grove, PA, USA) or anti-mouse (1:40,000; BD Biosciences) antibodies conjugated with horseradish peroxidase through the use of enhanced chemiluminescence reagents (ECL; Amersham Bioscience). β-actin (ACTB) was used as the internal control. We used Multi Gauge 3.0 software (LAS-4000) to estimate the expression of ERBB2, BIRC5 and CD99.

### Synthetic peptides

The research software BIMAS and data from the available literature were used to identify HLA-A*0201-specific peptides [28–30] (Table 1). Of the candidate HLA-A*0201-specific peptides, peptides were selected and synthesized for evaluation on the basis of high scores that predict HLA-A*0201 binding, as well as the presence of primary and secondary HLA-A*0201 anchor residues. The three selected peptides (BIRC5, CD99 and ERBB2) and their corresponding branched multipeptide were synthesized. All peptides were synthesized at PEPTRON (Daejeon, Korea). Mini-PEG spacers were used to design the branched multipeptide [21]. The purity of each synthetic peptide was confirmed to be >95% by reverse-phase high-performance liquid chromatography and mass spectrometry. Synthetic peptides were dissolved in dimethyl sulfoxide or distilled water, according to the manufacturer's recommendations, and stored at −20°C until use.

**Table 1:** Peptide sequence as predicted by BIMAS

Peptide	Start Position	Subsequence Residue Listing	Score
ERBB2	369	KIFGSLAFL	481
	106	QLFEDNYAL	324
	689	RLQETTELV	126
	789	CLTSTVQLV	159
BIRC5	5	TLPPAWQPEL	Reference [28]
	95	ELTGEFLKL	Reference [29]
	96 leucine	LLLGEFLKL	Reference [30]
	96 methionine	LMLGEFLKL	Reference [30]
CD99	9	LLLFGLLGV	1006
	6	ALALLLFGL	284
	13	GLLGVLVAA	42
	10	LLFGLLGVL	16

(Score: Estimate of half time of disassociation of a molecule containing this subsequence)

### Generation of DCs

Peripheral blood samples were collected from normal healthy donors carrying HLA-A*0201 after they had provided informed consent, according to the protocol approved by the Institutional Ethics Committee at the Chonnam National University Hwasun Hospital (CNUHH-2015-095). Monocytes were generated from peripheral blood mononuclear cells as previously reported [31]. For the generation of immature DCs, the monocytes were cultured in Iscove's Modified Dulbecco's Medium (Gibco-BRL) with 10% heat-inactivated FBS (Hyclone, Logan, UT, USA) and 1% PS for six days in 24-well plates at a density of 5 × 10^5^ cells per well with 100 ng/mL granulocyte macrophage colony-stimulating factor (LG Biochemical, Daejeon, Korea) and 20 ng/mL IL-4 (R&D Systems, Minneapolis, MN, USA). On day six, the immature DCs were matured through the addition of a type 1-polarized DC (αDC1) cocktail containing IL-1β (25 ng/mL), TNF-α (50 ng/mL), IFN-α (3,000 U/mL, Intron-A-IFN-α-2b; Schering–Plough), IFN-γ (1,000 U/mL; Strathmann Biotech) and Poly (I:C) (20 μg/mL; Sigma-Aldrich). The αDC1s were harvested on day eight.

### Peptide binding assay

The T2 cell line was used to evaluate the binding affinity of a multipeptide cocktail of the three selected HLA-A*0201 peptides - ERBB2_369_ (KIFGSLAFL), BIRC5_96methionine_ (LMLGEFLKL) and CD99_9_ (LLLFGLLGV). In brief, T2 cells were pulsed overnight with the multipeptide cocktail or branched multipeptide (at a total concentration of 0, 7.5, 15.0, 22.5, or 30.0 μg/mL) plus 3 μg/mL human β2-microglobulin (Sigma, USA). Following incubation, the cells were stained with an antihuman HLA-A2-FITCmAb (BB7.2; AbCam) and analyzed with a FACSCalibur flow cytometer (Becton Dickinson) and Win MDI version 2.9 (Bio-SoftNet). HLA-A2 expression was quantified according to the following formula: [(mean fluorescence with the peptide – mean fluorescence without the peptide) / mean fluorescence without the peptide] × 100. No peptide was used as a negative control, and WT-1_126_ (RMFPNAPYL) was used as an HLA-A2-specific positive control. The values represent the MFI. Results are reported as mean values ± SE of three separate experiments.

### Peptide stability assay

The multipeptide cocktail was examined for HLA-A2 stability. Briefly, T2 cells were pulsed overnight with the multipeptide cocktail or branched multipeptide (22.5 μg/mL) plus 3 μg/mL human β2-microglobulin. For the measurement of the peptide/HLA-A2 complex stability, cells were stained with BB7.2 at 0, 2, 4, 8 and 24 hours after treatment with BFA, and then were analyzed by flow cytometry. Results are reported as mean values ± SE of three separate experiments.

### Immunophenotyping of DCs

Phycoerythrin- or FITC-conjugated mAbs were added to the cell pellets, which were then incubated for 30 min on ice and washed three times before analysis. Mature αDC1s were stained with phycoerythrin-conjugated mAbs against CD80 and CD86. Moreover, DCs were stained with FITC-conjugated mAbs against CD83 and CCR7. DC phenotypes were assayed with a FACSCalibur flow cytometer, and the data were analyzed in Win MDI version 2.9. ERBB2, BIRC5 and CD99 individual peptide-pulsed αDC1s were termed as “A,B,C-pulsed αDC1s,” while mixed ERBB2, BIRC5 and CD99 peptide-pulsed αDC1s were termed as “A/B/C-pulsed αDC1s,” and branched multipeptide-pulsed αDC1s were termed as “Branched multipeptide-pulsed αDC1s” (A: ERBB2, B: BIRC5, C: CD99). Based on the isotype-matched negative controls, the results are reported as mean MFI values ± SE of three separate experiments.

### Cytokine analysis by enzyme-linked immunosorbent assay (ELISA)

The levels of IL-12p40, IL-12p70, IL-10 and IL-23 in the primary culture supernatants of DCs were measured with Quantikine Immunoassay Kits (R&D Systems). In addition, the DCs harvested on day eight were plated in 96-well plates at a density of 2 × 10^4^ cells per well, and cytokine secretion was stimulated through the use of CD40 ligand (CD40L)-transfected J558 cells (as an analog of CD40L-expressing Th cells; a kind gift from Dr. P. Lane, University of Birmingham, UK) at a density of 5 × 10^4^ cells per well. After 24 hours, the supernatant was harvested, and cytokine production was determined by ELISA. Each sample was analyzed in triplicate and the mean absorbance for each set of standards and samples was calculated.

### ELISPOT

The ELISPOT assay for the enumeration of antigen-specific IFN-γ-secreting cells was performed as indicated by the manufacturer (BD Biosciences).

We cultured effector CTLs, and either mixed them with ERBB2, BIRC5 and CD99 individual peptide-pulsed αDC1s and stimulated them with autologous CD3^+^ T cells (termed as “A,B,C DC-CTLs”); mixed them with ERBB2, BIRC5 and CD99 peptide-pulsed αDC1s and stimulated them with CD3^+^ T cells (termed as “A/B/C DC-CTLs”); or mixed them with branched multipeptide-pulsed αDC1s and stimulated them with CD3^+^ T cells (termed as “Branched DC-CTLs”).

T2 cells pulsed with the multipeptide cocktail were used as target cells for IFN-γ ELISPOT assay. These included T2 cells individually pulsed with the ERBB2, BIRC5 and CD99 peptides (termed as “A,B,C T2”) and T2 cells pulsed with mixed ERBB2, BIRC5 and CD99 peptides (termed as “A/B/C T2”). Branched multipeptide-pulsed T2 cells (termed as “Branched T2”) were also used as target cells. Unpulsed T2 cells (termed as “Unpulsed T2”) were used as a negative control, and HIV-1 gp160 (KLTPLCVTL)-pulsed T2 cells (termed as “HIV T2”) were used as an irrelevant control.

Further, the glioblastoma cell lines U87, U251 and U343 were used as target cells.

The number of IFN-γ spots was enumerated by an automatic CTL Immunospot Analyzer (Cellular Technology Ltd., Shaker Heights, OH, USA). All samples were run in triplicate, and the data are expressed as the mean number of spots ± SD per 10^5^ CD8^+^ T cells.

### LDH release cytotoxicity assay

The CytoTox 96 nonradioactive cytotoxicity assay (Promega, USA) was performed to measure the cytotoxic activity of CTLs according to the manufacturer's instructions. The effector cells were made with different combinations of the three peptides. Briefly, we cultured effector CTLs including A,B DC-CTLs, B,C DC-CTLs, A,C DC-CTLs, A,B,C DC-CTLs and Branched DC-CTLs. As for the target cells, U251 and primary glioblastoma cells (1×10^5^cells/well) were added to 96-well uncoated plates (Costar, USA). The effector CTLs were added at an E/T ratio of 10:1. The W6/32 monoclonal antibody (mAb; a gift from Dr. Bin Gao, ICH, London, United Kingdom) was used to block MHC class I antigen presentation on U251 and primary glioblastoma cells. Cells were washed twice with PBS and then incubated with the W6/32 mAb (1 μg/mL) on ice for one hour. The cells were then washed and used in cytotoxicity assays.

The spontaneous lactate dehydrogenase (LDH) release measurements of control groups of effector cells and target cells, the maximum LDH release measurements of target cells, the volume correction control and the culture medium background correction were performed at the same time. The plates were incubated for four hours in a humidified chamber at 37°C and 5% CO2, then centrifuged at 250 × *g* for five minutes. Aliquots (50 μL) were transferred from all wells to fresh 96-well flat-bottom plates, and an equal volume of reconstituted substrate mix was added to each well. The plates were incubated at room temperature for 30 min and protected from light. Then, 50 mL of stop solution was added, and the absorbance values were measured at 492 nm. The mean percentage of specific lysis in triplicate wells was calculated as follows: % Cytotoxicity = (Experimental - Effector Spontaneous - Target Spontaneous) / (Target Maximum - Target Spontaneous) × 100.

### Statistical analysis

All statistical analyses were performed with SPSS 13.0 for Windows (SPSS Inc., Chicago, IL, USA). One-way analysis of variance (ANOVA) was performed to analyze the statistical significance of non-parametric differences between the groups. P < 0.05 was considered statistically significant.
